# Validity, intra- and inter-observer reliability of automated devices for the assessment of ankle brachial index using photo-plethysmography

**DOI:** 10.1186/1471-2261-13-81

**Published:** 2013-10-08

**Authors:** Andrej Teren, Frank Beutner, Kerstin Wirkner, Markus Loeffler, Markus Scholz

**Affiliations:** 1 LIFE- Leipzig Research Center for Civilization Diseases, University of Leipzig, Leipzig, Germany; 2 Department of Internal Medicine/Cardiology, Heart Center of the University Leipzig, Leipzig, Germany; 3 Institute of Medical Informatics, Statistics and Epidemiology, LIFE, University of Leipzig, Haertelstrasse 16-18, 04107 Leipzig, Germany

**Keywords:** Ankle-brachial index, Feasibility, Vascular explorer, Vicorder

## Abstract

**Background:**

Ankle-brachial-Index (ABI) measured by manual Dopplersonography is an easily assessable marker of global cardiovascular risk. The aim of this study was to establish novel photo-plethysmography (PPG)-based ABI assessments in an epidemiologic context and to compare its results with those of Doppler.

**Methods:**

Two devices for PPG-based ABI assessments (Vicorder, Vascular Explorer) were tested and compared against Doppler in 56 putatively healthy subjects. We determined acceptance, time requirements, agreement of repeat measurements, agreement with Doppler and intra- and inter-observer concordances for both devices and compared the results. Differences between cuff inflation- and deflation-based methods were also studied for Vascular Explorer.

**Results:**

Acceptance was similar for both devices but Vascular Explorer was more time consuming. Agreement of multiple measurements was moderate for both methods highlighting the importance of measurement replicates. Both automated devices showed significantly higher ABI compared to Doppler which can be traced back to higher brachial pressures (Vicorder) or higher ankle pressures (Vascular Explorer). This effect is more pronounced for Vascular Explorer but can be ameliorated using the deflation method of measurement. Intra-observer concordances were similar. Inter-observer concordance was non-significantly better for Vicorder.

**Conclusions:**

Both devices proved to be feasible in epidemiologic studies, but compared to Doppler, do not constitute an advantage regarding time requirement and accuracy of ABI assessment. Since PPG-based ABI values are inflated compared to Doppler, it will be necessary to adjust Doppler-based cut-offs for risk stratification.

## Background

Ankle-Brachial Index (ABI) is an easily assessable diagnostic quotient noninvasively detecting the presence of peripheral artery disease (PAD). Several population-based studies demonstrated a high predictive value of ABI for global cardiovascular risk ([1], for Review see [2]). Therefore, it is of interest for epidemiologic research where it can be utilized as a screening tool for PAD or as risk factor for cardiovascular events.

Current guidelines consider the handheld *Doppler* sonography as the gold-standard for ABI determination [3]. However, a learning phase and ongoing training is required to obtain accurate and reliable results. In epidemiologic studies, assessments were usually performed by rotative technical staff requiring high standardization of assessments. Therefore, a number of alternative approaches for automated ABI-measurements were proposed, promising an easier handling, more standardized measurements and reduced observer variability.

*Vicorder* (SMT Medical Germany/Skidmore Medical UK) and *Vascular Explorer* (Enverdis, Germany) are two novel automatic devices allowing computation of ankle- and brachial pressures by software mediated analysis of photo-plethysmographic (PPG) signals from finger and toe. Despite of the similar techniques, the devices operate at separate protocols. *Vascular Explorer* (*VE*) determines ABI using blood pressures detected at disappearance of peripheral PPG signals during cuff inflation. In contrary, *Vicorder* (*VI*) records the pressure at the time point of reoccurrence of the PPG-pulse-wave during cuff deflation.

In order to introduce the optical PPG technology for ABI assessments in an epidemiologic context, we performed a feasibility study aiming to estimate the intra- and inter-observer concordance as well as the agreement of the PPG-derived results with *Doppler* sonographic ABI measurements. Furthermore, we evaluated the feasibility of automated measurements with respect to time requirements and compliance. Finally, we developed standard operating procedures (SOP) for all three devices.

Results of our study served as a basis of decision-making for selecting a method of ABI assessment for our own ongoing population based epidemiologic study (LIFE) planning to recruit several thousand individuals.

## Methods

### Study sample

Repeated ABI measurements were performed in a convenience sample of 56 subjects selected from the pilot survey of the LIFE Project (Leipzig Research Center for Civilization Diseases). Exclusion criteria were critical limb ischämia, ulcera cruris, angina pectoris, heart insufficiency, lymphoedema and paraneoplastic lymphadenopathy. All individuals negated claudication, confirmed diagnose of peripheral artery disease or history of peripheral revascularization. Study sample comprises 23 males and 33 females of median age = 69.5 years (interquartile range (IQR) = 65.0-72.0 years), median BMI = 27.4 kg/m^2^ (IQR = 25.4-30.7 kg/m^2^). In 26 subjects we observed a systolic blood pressure >135 mmHg based on *Doppler* sonography, averaged over the three measurements. All subjects included in the study gave their written informed consent. The study was approved by the ethical committee of the Medical Faculty of the University of Leipzig.

### ABI determination

Observers were experienced in *Doppler*-based ABI determination. In preparation for this study, all observers were trained for both automated devices according to the instructions of the manufacturers and own standard operating procedures (in German, available upon request). Technical handling was practiced in a training sample of 20 probands prior to the study. Details of the measurements with *Doppler* and automated measurements can be found elsewhere [4].

Subjects were placed in a supine position for at least 10 minutes before starting the measurements. First, systolic blood pressures of the right arm and both ankles (A. tibialis posterior) were measured with standard *Doppler* method using sphygmomanometer cuffs and a handheld *Doppler* probe (Huntleigh Mini-Dopplex, Luton, UK) [5]. Subsequently, measurements with *Vicorder* (Software Version 4.3.3898.19242) and *Vascular Explorer* (Software Version 2.0.5) were performed according to the study protocol (see next section).

**Figure 1:**
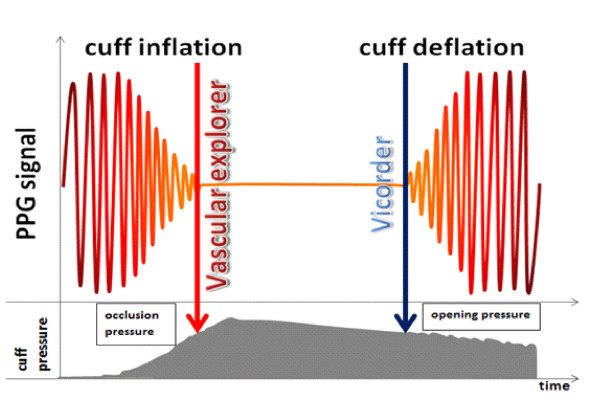
**Illustration of ABI determination by Vicorder and Vascular Explorer.** We present the default methods of both PPG-based devices. While Vascular Explorer measures during the disappearance of signal (inflation method), Vicorder measures during the re-appearance of the signal (deflation method).

Additional manual corrections of automatically measured blood pressures obtained by *Vicorder* or *Vascular Explorer* were performed at the discretion of the examiner immediately after each measurement and by an experienced supervisor after completion of the study. All corrections were based on an optional displacement of the measuring trace to an area of the curve which visually more likely correspond to PPG signal loss or reappearance respectively depending on the approach currently being considered (inflationary vs. deflationary, see Figure 1).

Automatically assessed findings and observer-revised findings were stored separately. Thus, measures are defined as automatic default settings (*Vicorder*, *Vascular Explorer*) and observer-revised values (*observer*/*examiner revised Vicorder*, *observer*/*examiner revised Vascular Explorer*). Additionally, in *Vascular Explorer* which detects disappearance of the PPG signal during the cuff inflation by default, the deflationary method was recorded manually and stored separately (*deflation method of Vascular Explorer*). For each application of one of the devices, measurements were repeated three times directly in a row.

After the measurements, subjects were requested to evaluate the acceptability of both automated devices in order to get an estimate of the compliance. Acceptability was measured on a discrete scale of 1 (worst) to 5 (best). We also determined the time requirements necessary for the measurements with the automated devices. This was assessed on the basis of the complete measurement process including application of cuffs and three measurement replicates of both, blood pressures and pulse wave parameters not considered in the present work. Time requirements for undressing of probands were not included here.

### Study design

The study design is presented in Table 1. The first four series are designed to assess the inter-observer concordance since both devices are used by two different trained medical technical assistants at the same patients. The sequence of the measurements was shuffled between series 1 to 4 in order to avoid biases due to order effects. Analogously, series 5 to 8 are design to assess the intra-observer concordance. Subjects were randomly assigned to the series. The same two observers (A/B) were used for all assessments.

**Table 1:** Study design

**Serial number**	**Assessment plan**					**No. of probands**
1	D-A	VE - A	VI - A	VE – B	VI – B	7
2	D-A	VI - A	VE - A	VI – B	VE – B	7
3	D-B	VE – B	VI - B	VE - A	VI – A	7
4	D-B	VI – B	VE – B	VI - A	VE – A	7
5	D-A	VE - A	VI - A	VE - A	VI – A	7
6	D-A	VI - A	VE - A	VI - A	VE – A	7
7	D-B	VE - B	VI - B	VE - B	VI – B	7
8	D-B	VI - B	VE - B	VI - B	VE – B	7

Assessments were performed in the prescribed consecutive order. D = Doppler sonography, VI = Vicorder, VE = Vascular explorer, A,B = different observers.

### Statistical analysis

ABI measurements were analysed per limb, i.e. right side and left side ABI measurements were treated as independent throughout. The following questions were addressed:

1. Reliability of repeated measurements, i.e. within-set agreement of ABI-triplicates (*Doppler*, *Vascular Explorer*, *Vicorder*). This analysis is based on the first measurement triplicate of each device.

2. Agreement of automated ABI results of *Vascular Explorer* and *Vicorder* with *Doppler*. This analysis is based on the ABI measurements averaged over the first measurement triplicate of each device.

3. Inter-observer reliability of averaged ABI measurements of *Vicorder*, *Vascular Explorer*) based on the series 1–4 of our assessment plant (see Table 1).

4. Intra-observer reliability of averaged ABI measurements of *Vicorder*, *Vascular Explorer*) based on the series 5–8 of our assessment plant (see Table 1).

5. Impact of manual correction (revised *Vicorder* and *Vascular Explorer*, *Vascular Explorer*’*s deflation method*) on within-set agreement, agreement with *Doppler*, intra- and inter-observer reliability.

6. Feasibility of automated ABI assessments in population-based epidemiologic studies.

Agreement of measurements was evaluated using concordance correlation coefficients (CCC). The CCC was recommended by Lin for assessing agreement of continuous variables [6]. Its values range between -1 and 1. Maximum is achieved if and only if the mean difference between the samples is zero, the correlation is 1 and the variances are the same. The CCC can be generalized to more than two measurements to be compared (overall concordance correlation coefficient – OCCC, see [7]). We calculated confidence bounds of the measure by estimating Jack-Knife standard errors of the Fisher-transformed CCC in analogy to [8]. Similarly, we constructed formal statistical tests of the differences of two CCC based on the same samples by estimating Jack-Knife standard errors of the difference of two CCCs.

Biases of methods are estimated by arithmetic means of differences of paired data (e.g. first versus second measurement of a method, mean of *Doppler* triplicates versus mean of *Vicorder* triplicates). Significances of biases were calculated by one-group t-Tests. Analogously, biases were compared between methods using paired t-Tests of individual differences (e.g. differences *Vicorder* - *Doppler* versus differences *Vascular Explorer* - *Doppler*).

For graphical assessments we construct scatter plots and Bland-Altman plots. Some of the analyses were also performed for the raw blood pressure measurements at arm and ankle in order to determine potential sources of observed biases of ABI measurements. Duration of assessments and satisfaction of probands were compared between the automated methods using the Wilcoxon signed rank sum test.

In view of the exploratory nature of the study presented, we performed no corrections of multiple testing. Hence our significances must be considered as 'suggestive’ but not 'evidentiary’.

All analyses were implemented and performed using the statistical software package R 2.13.1 (http://www.r-project.org).

## Results

### Feasibility

Acceptance was measured as score between 1 (worst) to 5 (best). Mean of acceptance score was 4.30 for *Vicorder* (Score 1: N = 2, Score 2: N = 5, Score 3: N = 2, Score 4: N = 12, Score 5: N = 35). *Vascular Explorer* received a mean acceptance score of 4.36 (Score 1: N = 2, Score 2: N = 3, Score 3: N = 3, Score 4: N = 13, Score 5: N = 35). The difference was not significant (p = 0.67). Median duration of measurements was 19 min (IQR = 17-21 min) for *Vicorder* and 22 min (IQR = 20-24 min) for *Vascular Explorer* (p < 0.001).

### Concordance of repeated measurements

This analysis is based on the first measurement triplicates performed for the assessments. Analyses revealed different concordances of repeated ABI measures between the three techniques. For *Doppler* we observed the best within-set concordance of triplicates (OCCC 0.88; 95%-CI 0.83-0.92). Pair-wise concordance did not differ significantly between first and second, first and third or second and third measurement. *Vicorder* measurements showed a better overall concordance compared to *Vascular Explorer* (OCCC 0.69; 95%-CI 0.58–0.78 vs. OCCC 0.49; 95%-CI 0.39–0.58, p = 0.060). The pair-wise concordances were similar for *Vicorder* and *Vascular Explorer* measurements.

Concordance of measurements was improved by the correction methods. At this, examiner revised methods performed best, resulting in significantly better concordances than the uncorrected values (*Vicorder*: OCCC 0.80; 95%-CI 0.74 – 0.84 (p = 0.005), *Vascular explorer*: OCCC 0.67; 95%-CI 0.60 – 0.74 (p = 0.004)). After correction, the concordance of *Vicorder* measurements is still superior to that of *Vascular explorer* (p = 0.0028). *Deflation method of Vascular Explorer* performed similarly compared to the raw values of *Vascular Explorer* (OCCC 0.54; 95%-CI 0.43 – 0.63 (p = 0.31)). Concordances of ankle and brachial pressures were comparable for each device and correction method (see Additional file 1: Table S1).

### Agreement of automated PPG-based measurements with Doppler

This analysis is based on all measurement series of our assessment plan. For each device, we calculated the average of the first measurement triplicate.

**Figure 2:**
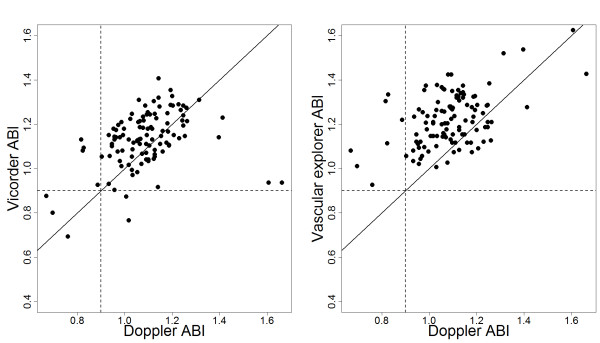
**Agreement of automated ABI measurements those of Doppler sonography.** We present the raw data of both devices. Both automated ABI measurements are clearly biased towards higher values which is more pronounced for Vascular Explorer.

We observed only a moderate concordance with *Doppler* for both automated devices (OCCC: *Vicorder* 0.34 vs. *Vascular Explorer* 0.33, p = 0.95, Figure 2). Both automated methods resulted in a significant bias towards higher ABI values which is especially pronounced for the uncorrected measurements of *Vascular Explorer* (*Vicorder* +0.05, *Vascular Explorer* +0.13 (p < 0.001)).

Correction methods only marginally improved the concordances for both devices. *Deflation method of Vascular explorer* showed the smallest bias of Vascular Explorer variants which was significantly smaller than that of the uncorrected values (p < 0.001) but still significantly higher than that of *Vicorder* (p = 0.0042).

Concordances of brachial pressures were better than for ankle pressures. Compared to *Doppler*, we observed a significantly negative bias of brachial pressures for *Vicorder* methods and a strong positive bias of ankle pressures for *Vascular Explorer* methods. This explains the upward bias observed for both devices. Detailed results can be found in Additional file 1: Table S2.

### Intra-observer concordance

**Figure 3:**
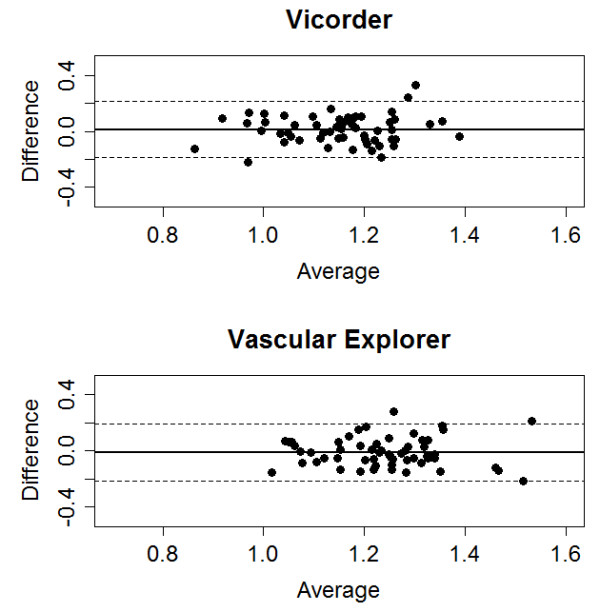
**Bland-Altman plots for intra-observer agreement.** We present the results of the raw ABI measurements for both Vicorder and Vascular Explorer.

Series 5–8 were designed to estimate the intra-observer concordance of the automated methods. *Vicorder* and *Vascular Explorer* showed comparable, moderate to good intra-observer concordance and no significant bias between first and second measurement (differences of CCC: p = 0.92, differences of bias: p = 0.18, Table 2). Correction methods of *Vicorder* resulted in essentially the same concordances than the standard measurements. In contrast, *observer*/*examiner revised Vascular explorer* improved concordance while the *deflation method of Vascular Explorer* decreased concordance slightly compared to standard measurements. Nevertheless, none of the differences reached significance. Bland-Altman plots of the raw values are shown in Figure 3.

**Table 2:** Intra-observer concordances of ABI measurements

**Method**	**CCC**	**95%-CI of CCC**		**Bias**	**p-value**	**Correlation**
VI	0.65	0.46	0.78	0.015	0.29	0.65
VI_O	0.68	0.51	0.80	0.001	0.95	0.68
VI_E	0.65	0.47	0.78	0.006	0.65	0.66
VE	0.65	0.50	0.76	-0.011	0.43	0.66
VE_O	0.75	0.59	0.85	-0.004	0.75	0.75
VE_E	0.76	0.57	0.87	-0.002	0.89	0.76
VE_D	0.57	0.27	0.78	0.021	0.12	0.59

Agreement of first and second ABI measurement for automated methods. We present concordance correlation coefficients, corresponding confidence intervals, bias between first and second measurements (values less than zero mean lower values at first measurement), p-value of bias and correlation coefficient of first and second measurement.

VI/VE = Vicorder/Vascular Explorer standard measurements, VI_O/VE_O = Vicorder/Vascular Explorer corrected by observer, VI_E/VE_E = Vicorder/Vascular Explorer corrected by examiner, VE_D = deflation method of Vascular Explorer.

Analysis of brachial and ankle blood pressures revealed a bias towards lower blood pressures at the second measurement compared to the first measurement which is more pronounced for the measurements based on *Vicorder* (e.g. *Vicorder* brachial: -5.2 mmHg, ankle: -7.5 mmHg, *Vascular Explorer*’*s inflationary method* brachial: -4.6 mmHg, ankle: -4.4 mmHg). Intra-observer concordances of blood pressure measurements were similar for the devices and correction methods (see Additional file 1: Table S3 for further details).

### Inter-observer concordance

**Figure 4:**
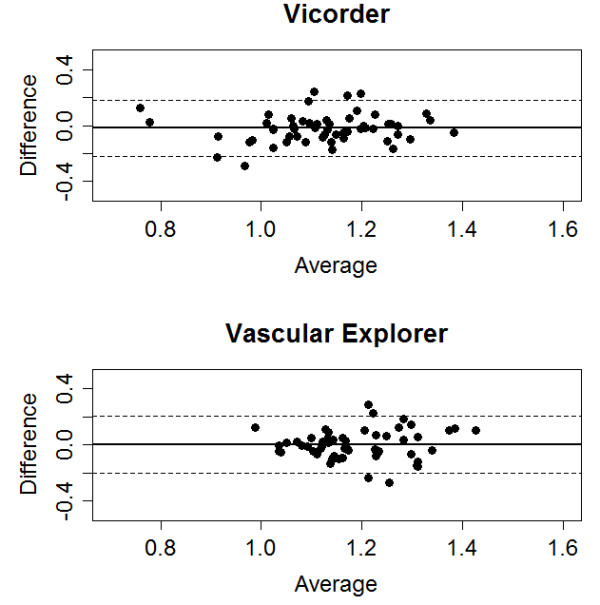
**Bland-Altman plots for inter-observer agreement.** We present the results of the raw ABI measurements for both Vicorder and Vascular Explorer.

Series 1–4 were designed to estimate the inter-observer concordance of the automated methods. Raw values of *Vicorder* showed superior inter-observer concordance compared to *Vascular explorer* but the difference is not significant (p = 0.22, Table 3). Both methods showed no significant bias between observers (p = 0.18 vs. p = 0.98, difference of bias: p = 0.31). None of the correction methods improved the concordances significantly. Bland-Altmann plots of raw values can be found in Figure 4.

**Table 3:** Inter-observer concordances of ABI measurements

**Method**	**CCC**	**95%-CI of CCC**		**Bias**	**p-value**	**Correlation**
VI	0.70	0.50	0.83	-0.019	0.18	0.71
VI_O	0.71	0.51	0.84	-0.032	0.017	0.73
VI_E	0.73	0.53	0.85	-0.026	0.046	0.74
VE	0.55	0.31	0.73	-0.00042	0.98	0.55
VE_O	0.49	0.23	0.69	-0.010	0.46	0.50
VE_E	0.54	0.21	0.76	-0.024	0.060	0.55
VE_D	0.56	0.16	0.89	-0.023	0.16	0.58

Agreement of first and second ABI measurement for automated methods. We present concordance correlation coefficients, corresponding confidence intervals, bias between observers, p-value of bias and correlation coefficient between observers.

VI/VE = Vicorder/Vascular Explorer standard measurements, VI_O/VE_O = Vicorder/Vascular Explorer corrected by observer, VI_E/VE_E = Vicorder/Vascular Explorer corrected by examiner, VE_D = Deflation method of Vascular Explorer.

Results of concordance analyses of brachial and ankle pressure are presented in Additional file 1: Table S4. While the raw values of *Vicorder* expressed no bias between observes, there was a small significant difference for *Vascular Explorer* which points into the same direction for ankle and brachial pressures. The correction methods did not improve these observations.

## Discussion

ABI determination is useful for the assessment of peripheral vessel status and cardiovascular risk in epidemiologic studies. New automated tools based on photo-plethysmography were established which can simultaneously measure ABI and other vascular parameters such as pulse wave velocity. These devices promise an easier handling compared to *Doppler* sonography which could improve the standardization of these assessments in large epidemiologic trials. In the present study, we aimed at introducing plethysmography-based ABI assessments in an epidemiologic context by assessing feasibility, agreement with *Doppler* sonography, intra- and inter-observer concordances. We compared the results between the two devices *Vicorder* and *Vascular Explorer* whose measurements are based on the deflation and inflation method respectively.

We also analysed the performance of a number of correction methods of the raw values of *Vicorder* and *Vascular Explorer*, namely observer or examiner revised values for both methods and the *deflation method of Vascular Explorer*. The latter one showed better agreement with *Doppler* especially for patients with peripheral artery disease [4].

Acceptance scores of both devices are similarly high. Moreover, we documented a comparably short learning and training phase for both devices and observers, including the training for manual ABI corrections. Therefore, we conclude that both assessments are feasible in large population based studies. However, measurements with *Vascular Explorer* were more time-consuming mainly due to multiple inflations of the ankle cuff in case of low signal quality. This applies in particular for subjects with high blood pressure and might be preventable by appropriate software modifications. Considering the economic aspect, the costs for both PPG-devices in our hands were comparable (*Vicorder*: about 13,000€, *Vascular Explorer*: about 11,000€) but an order of magnitude higher than for hand-held Doppler (below 1,000€).

While measurement triplicates of *Doppler* express high concordance, it was considerably lower for the automated measurements. The high concordance of the repeated Doppler measurement could be attributed to the subjective evaluation of the acoustic Doppler signal. Accordingly, after visual correction of the raw ABI values, both PPG-based devices improved their within-set concordances. Deflation modus in *Vascular Explorer* did not improve the within-set concordance indicating that the differences between measurement replicates are not modus-specific. We conclude that at least three repeated measurements are required in an epidemiologic study to control the variability of automated ABI measurements.

In the present study, we observed a strong bias of automated methods towards higher ABI values compared to *Doppler*. The bias is especially pronounced for *Vascular Explorer*. The observed bias is caused by either a negative bias of brachial pressures (*Vicorder*) or a positive bias for ankle pressures (*Vascular Explorer*). The negative bias for brachial pressures is in accordance to findings reported by Jobbagy et al. [9] who demonstrated a substantial delay in PPG detection of artery opening during the cuff deflation at the upper arm when compared with deflationary oscillometry performed by OMRON M4. Higher ankle pressures during automated PPG-based measurement can be attributed to the assessment of overall limb circulation, whereas *Doppler* detects only one particular artery of interest. Accordingly, a possible sustained leg perfusion through collaterals might be a source of higher ABI-values. Moreover, the higher biases observed for the *Vascular Explorer* could be a result of the prolonged tibial artery occlusion times due to frequently longer or repeated ankle cuff inflations mentioned above. The temporary increase of systolic and diastolic blood pressure as reaction on the cuff inflation is well documented especially in subjects with hypertension [9]. Similarly, we observed reactive pressure increase by *Vicorder*, however, almost exclusively at the first measurement. Thus, further improvement of the current sensors and detection algorithms might avoid the redundant cuff inflations which could improve the measurements for the inflationary methods in the future.

The *deflationary method of Vascular Explorer* ameliorated the observed bias. However, it was still significantly larger than for *Vicorder*. We showed in [4] that the *deflation method of Vascular explorer* works especially well for improving the concordance of low ABI values which are less common in our study population. *Vicorder* and *Vascular Explorer* showed good intra- and inter-observer concordances. Intra-observer concordances appeared to be virtually identical. In contrast, the inter-observer concordance was (non-significantly) better for *Vicorder*.

For both devices, observer or examiner based corrections of raw values failed to provide clearly better results with respect to agreement with *Doppler* or intra- and inter-observer concordances. Therefore, we recommend using the raw values. In our study, and on the basis of our SOPs and Software Versions, *Vicorder* performed slightly better with respect to time requirements, agreement with Doppler, variance of repeated measurements and inter-observer agreement. Therefore, we decided to use *Vicorder* in our population based cohort aiming at recruiting 10,000 individuals (LIFE study). However, since both, Software Version and SOPs can be subject to changes, our finding should not be over-interpreted in the sense that *Vascular Explorer* is generally inferior.

Compared to *Doppler* both PPG-based methods do not constitute an advantage regarding time requirement and accuracy of ABI assessment. However, a major potential of PPG-based methods is the availability of other traits of vessel status such as pulse-wave parameters. Hence, the additional time requirement might be justified by a deeper characterization of individual’s vessel status. Since PPG-based ABI values are inflated compared to Doppler, it will be necessary to adjust Doppler-based cut-offs for risk stratification. We could show that proper adjustments of corresponding cut-offs could result in the same diagnostic power to detect PAD as *Doppler* based ABI. But definite recommendations for adjusted cut-offs would require larger sample sizes of both, PAD patients and controls [4].

There are several limitations of our study: We analysed a convenience sample which could imply that we over-estimated the acceptance but under-estimated the time requirements compared to a population-based sample. We used *Doppler* sonography as gold-standard since invasive blood pressure measurements and angiography are ethically not justified for our putatively healthy individuals. The sample size of our study is limited in view of the extensive assessment programme required to answer our questions. This did not allow us to estimate concordances and biases with high accuracy. Additionally, due to the same reason, we neither performed any sub-group analyses nor analyses of the effects of covariates on the performance of methods. Finally, we like to emphasize again that our results depend on the actual hard- and software versions of the devices and our SOPs. Since there are ongoing activities regarding improvements, it will be necessary to repeat our study if new versions are available. However, we believe that our study provides a first rationale for the application of automated PPG-based methods for vascular assessments in large epidemiologic studies.

## Conclusion

Both PPG-based methods proved to be feasible in epidemiologic studies, but compared to Doppler, do not constitute an advantage regarding time requirement and accuracy of ABI assessment. Since PPG-based ABI values are inflated compared to Doppler, it will be necessary to adjust Doppler-based cut-offs for risk stratification. Intra- and inter-observer concordances of PPG-based methods are satisfying, but three replicates are required to capture the variance of repeated measurements.

## Abbreviations

ABI: Ankle brachial index; CCC: Concordance correlation coefficient; IQR: Inter-quartile range; PAD: Peripheral artery disease; PPG: Photo-plethysmography; OCCC: Overall concordance correlation coefficient (CCC for more than two groups); SOP: Standard operating procedure; VE: Vascular explorer; VI: Vicorder.

## Competing interests

The authors declare that they have no competing interests.

## Authors’ contributions

AT performed the study and revised the manuscript, FB contributed to paper writing, KW performed the study, ML contributed to discussion, MS design the study, analysed the data and wrote the manuscript. All authors read and approved the final manuscript

## Pre-publication history

The pre-publication history for this paper can be accessed here:

http://www.biomedcentral.com/1471-2261/13/81/prepub

## Supplementary Material

Additional file 1 **Supplementary Results.** This file contains additional tables with analysis results. Click here for file
