# Supplementary material for: Validity, intra- and inter-observer reliability of automated devices for the assessment of ankle brachial index using photo-plethysmography
Source: BMC Cardiovasc Disord. 2013 Oct 8;13:81. doi: 10.1186/1471-2261-13-81 (PMC3851735; doi:10.1186/1471-2261-13-81)
Supplement: Additional file 1 — Supplementary Results. This file contains additional tables with analysis results. [file 1471-2261-13-81-S1.pdf]

Supplementary Results

Supplemental table S1:

Concordance correlation coefficients of repeated measurements and corresponding confidence intervals separated for ABI, brachial and ankle blood pressures

D = Doppler sonography,

VI / VE = Vicorder / Vascular explorer standard measurements,

VI_O / VE_O = Vicorder / Vascular explorer corrected by observer,

VI_E / VE_E = Vicorder / Vascular explorer corrected by examiner,

VE_D = Vascular explorer deflation method

Group	Method	OCCC	95% confidence interval of OCCC	
ABI	D	0.88	0.83	0.92
	VI	0.69	0.58	0.78
	VI_O	0.77	0.71	0.82
	VI_E	0.80	0.74	0.84
	VE	0.49	0.39	0.58
	VE_O	0.62	0.55	0.69
	VE_E	0.67	0.60	0.74
	VE_D	0.54	0.43	0.63
brachial	D	0.94	0.92	0.96
	VI	0.82	0.72	0.89
	VI_O	0.91	0.88	0.92
	VI_E	0.93	0.90	0.94
	VE	0.70	0.63	0.75
	VE_O	0.80	0.75	0.85
	VE_E	0.83	0.78	0.86
	VE_D	0.81	0.76	0.85
ankle	D	0.95	0.92	0.96
	VI	0.89	0.85	0.92
	VI_O	0.90	0.88	0.93
	VI_E	0.91	0.88	0.93
	VE	0.70	0.62	0.76
	VE_O	0.80	0.75	0.85
	VE_E	0.83	0.77	0.87
	VE_D	0.80	0.74	0.84

Supplemental table S2:

Agreement of automated methods with Doppler sonography ABI, brachial and ankle blood pressures

VI / VE = Vicorder / Vascular explorer standard measurements,

VI_O / VE_O = Vicorder / Vascular explorer corrected by observer,

VI_E / VE_E = Vicorder / Vascular explorer corrected by examiner,

VE_D = Vascular explorer deflation method

Positive bias indicates higher values of automated methods compared to Doppler sonography

Group	Method	CCC	95% CI of CCC		bias	p.value	correlation
ABI	VI	0.34	0.053	0.58	0.047	0.0016	0.36
	VI_O	0.41	0.18	0.60	0.053	<0.001	0.44
	VI_E	0.42	0.21	0.59	0.050	<0.001	0.46
	VE	0.33	0.16	0.48	0.133	<0.001	0.50
	VE_O	0.37	0.16	0.55	0.110	<0.001	0.50
	VE_E	0.36	0.18	0.52	0.104	<0.001	0.50
	VE_D	0.33	0.13	0.50	0.084	<0.001	0.40
brachial	VI	0.71	0.57	0.81	-4.17	0.012	0.73
	VI_O	0.76	0.64	0.85	-4.93	<0.001	0.80
	VI_E	0.77	0.65	0.85	-4.23	0.0039	0.80
	VE	0.69	0.54	0.80	-1.70	0.29	0.71
	VE_O	0.74	0.61	0.83	-0.93	0.55	0.75
	VE_E	0.79	0.68	0.86	1.37	0.33	0.80
	VE_D	0.74	0.61	0.83	-0.82	0.60	0.75
ankle	VI	0.59	0.34	0.76	1.40	0.45	0.60
	VI_O	0.63	0.43	0.78	1.48	0.40	0.64
	VI_E	0.63	0.45	0.77	1.82	0.29	0.64
	VE	0.54	0.41	0.64	15.33	<0.001	0.71
	VE_O	0.59	0.46	0.70	13.00	<0.001	0.72
	VE_E	0.51	0.36	0.63	15.73	<0.001	0.67
	VE_D	0.59	0.46	0.70	12.92	<0.001	0.72

Supplemental table S3:

Intra-observer agreement of automated blood pressure measurements

VI / VE = Vicorder / Vascular explorer standard measurements,

VI_O / VE_O = Vicorder / Vascular explorer corrected by observer,

VI_E / VE_E = Vicorder / Vascular explorer corrected by examiner,

VE_D = Vascular explorer deflation method

Positive bias indicates higher values at the first measurement compared to the second measurement

Group	Method	CCC	95% CI of CCC		bias	p.value	correlation
brachial	VI	0.80	0.70	0.87	5.24	0.0014	0.83
	VI_O	0.79	0.69	0.86	6.34	<0.001	0.83
	VI_E	0.79	0.70	0.86	5.60	<0.001	0.83
	VE	0.71	0.53	0.82	4.60	0.0022	0.75
	VE_O	0.81	0.66	0.90	3.45	0.010	0.83
	VE_E	0.81	0.65	0.90	3.53	0.012	0.82
	VE_D	0.80	0.63	0.90	3.39	0.014	0.82
ankle	VI	0.78	0.65	0.86	7.51	<0.001	0.82
	VI_O	0.79	0.68	0.87	7.19	<0.001	0.84
	VI_E	0.77	0.66	0.84	6.92	<0.001	0.81
	VE	0.80	0.68	0.87	4.42	0.0088	0.83
	VE_O	0.82	0.72	0.89	4.11	0.014	0.85
	VE_E	0.85	0.77	0.90	3.29	0.030	0.87
	VE_D	0.81	0.71	0.88	4.36	0.010	0.85

Supplemental table S4:

Inter-observer agreement of automated blood pressure measurements

VI / VE = Vicorder / Vascular explorer standard measurements,

VI_O / VE_O = Vicorder / Vascular explorer corrected by observer,

VI_E / VE_E = Vicorder / Vascular explorer corrected by examiner,

VE_D = Vascular explorer deflation method

Group	Method	CCC	95% CI of CCC		bias	p.value	correlation
brachial	VI	0.51	0.21	0.72	2.69	0.13	0.52
	VI_O	0.65	0.44	0.80	3.74	0.0087	0.68
	VI_E	0.68	0.48	0.81	3.17	0.020	0.70
	VE	0.69	0.54	0.80	-3.25	0.020	0.71
	VE_O	0.68	0.52	0.80	-4.15	0.0034	0.72
	VE_E	0.74	0.61	0.83	-1.01	0.46	0.75
	VE_D	0.69	0.53	0.81	-4.21	0.0028	0.73
ankle	VI	0.71	0.59	0.80	0.87	0.62	0.73
	VI_O	0.70	0.57	0.79	0.31	0.86	0.71
	VI_E	0.69	0.55	0.79	0.45	0.81	0.71
	VE	0.62	0.42	0.76	-3.94	0.019	0.64
	VE_O	0.55	0.36	0.70	-6.48	<0.001	0.61
	VE_E	0.56	0.36	0.72	-4.63	0.011	0.59
	VE_D	0.53	0.32	0.69	-6.17	0.0012	0.58
